# Repurposing epetraborole to combat *Neisseria gonorrhoeae* and *Chlamydia trachomatis* infections

**DOI:** 10.1128/aac.01445-25

**Published:** 2025-12-05

**Authors:** Abdallah S. Abdelsattar, Babatomiwa Kikiowo, Nader S. Abutaleb, Autumn S. Dove, Mohamed N. Seleem

**Affiliations:** 1 Department of Biomedical Sciences and Pathobiology, Virginia-Maryland College of Veterinary Medicine, Virginia Polytechnic Institute and State University 229659, Blacksburg, Virginia, USA; 2 Center for One Health Research, Virginia Polytechnic Institute and State University 1757, Blacksburg, Virginia, USA; University of Houston, Houston, Texas, USA

**Keywords:** epetraborole, sexually transmitted infections, antibacterial resistance, inclusion bodies, bacterial intracellular clearance, *In vivo *mouse model

## Abstract

*Neisseria gonorrhoeae* and *Chlamydia trachomatis* are the most common bacterial sexually transmitted infections. The World Health Organization (WHO) estimates that there were approximately 211 million cases of *chlamydia* and *gonorrhea* in 2020. Currently, no single drug is effective against both pathogens. Ceftriaxone (CRO) is the only recommended treatment for gonococcal infections but has no activity against *C. trachomatis*. Azithromycin (AZM) or doxycycline (DOX) is recommended for *C. trachomatis* infections; however, *N. gonorrhoeae* has developed resistance to both agents. Without new therapeutic options, these infections risk becoming untreatable. Utilizing a drug repurposing approach, we identified epetraborole (EBO) as a potent inhibitor for *N. gonorrhoeae* and *C. trachomatis*. EBO is a boron-containing compound currently in clinical trials for the treatment of non-tuberculous mycobacterial infections. EBO demonstrated potent activity against multidrug-resistant *N. gonorrhoeae* with MIC ranging from 0.125 to 0.25 µg/mL. Additionally, EBO exhibited anti-*C*. *trachomatis* activity at concentrations of ≤1 µg/mL. Furthermore, EBO was capable of eliminating the intracellular burden of both *N. gonorrhoeae* and *C. trachomatis,* surpassing the activity of CRO and AZM. Moreover, unlike CRO and AZM, EBO showed limited activity against the normal vaginal microbiota. Finally, in an *in vivo* mouse model of CRO-resistant *N. gonorrhoeae* genital tract infection, EBO exhibited a 99.95% reduction in bacterial burden after 2 days of treatment. Collectively, our findings highlight EBO as a promising candidate for the treatment of sexually transmitted infections that warrants further investigation.

## INTRODUCTION

Sexually transmitted infections (STIs) significantly affect sexual and reproductive health worldwide and can lead to severe complications, such as genital ulcers, maternal death, infertility, endocarditis, lower abdominal pain, arthritis, pelvic inflammatory disease, and urethral, vaginal, or anorectal discharge (1–5). According to the World Health Organization (WHO), more than 1 million people aged 15 to 49 years require treatment for STIs every day (6). *Chlamydia trachomatis* and *Neisseria gonorrhoeae* are the most prevalent bacterial STIs, with an estimated 129 and 82 million new cases, respectively, in 2020 (7, 8).

*C. trachomatis* is an obligate intracellular bacterium with two established phases, non-replicating elementary bodies that attach and initiate the infection, and reticulate bodies that replicate and form inclusions (9–11). Once internalized, the elementary body differentiates into the reticulate body, which then replicates inside the host cell, forming an inclusion. These new reticulate bodies will eventually undergo secondary differentiation into elementary bodies, which can infect other cells (12, 13). *C. trachomatis* infections are often treated with the broad-spectrum antibiotics doxycycline (DOX), azithromycin (AZM), and levofloxacin. Yet, reports of treatment failures have increased, including reports of repeated infections (14–19). For instance, the efficacy rate of AZM against rectal *Chlamydia* infections in men is only 74% (20), and the re-infection rate for *C. trachomatis*-infected women treated with AZM ranges from 18% to 34% (21, 22). Furthermore, *C. trachomatis* can develop mutations in the 23S rRNA, inducing resistance to AZM (23). For these reasons, in 2020, the Centers for Disease Control (CDC) changed the treatment guidelines from using AZM to using DOX for treating uncomplicated genitourinary chlamydia (14, 24). The use of levofloxacin is compromised by the increasing rate of development of resistance, and it has shown less potency than AZM and DOX (25, 26). Additionally, repeated use of the anti-*C*. *trachomatis* agents disturbs the composition of the vaginal microbiota which serves as the primary line of defense against STIs (27). Thus, providing novel therapies against *Chlamydia* is essential to reduce the pathogen’s prevalence.

*N. gonorrhoeae* is a bacterial pathogen that mostly causes infections in the genitals, rectum, and throat (28). Recently, according to the new guidelines of the CDC, the treatment regimen for gonorrhea was modified by removing AZM, leaving injectable ceftriaxone (CRO) as the last remaining treatment with no effective oral options available (24, 29, 30). However, resistance to CRO has been reported globally (31–34). Most seriously, *N. gonorrhoeae* isolates exhibiting combined resistance to AZM and CRO have been documented worldwide (35–37). Consequently, the CDC has categorized *N. gonorrhoeae* as an urgent threat that necessitates the development of new therapies (24, 29, 38, 39).

Further compounding the problem, the co-infection rates of chlamydia and gonorrhea are estimated between 50% and 70%. Women with gonorrhea are co-infected with chlamydia in 17.6%–57.9% of cases, while chlamydia-infected women are co-infected with gonorrhea in 2.1%–17.2% of cases (40–43). Moreover, during co-infection, *N. gonorrhoeae* induces *Chlamydia* to enter a difficult-to-treat persistence-like state (44). Furthermore, it was reported that treatment with antibiotics non-specific to *C. trachomatis* can lead to increasing rates of *N. gonorrhoeae* resistance (45–47). Treating co-infections can be challenging because the drugs that are effective against chlamydia are ineffective against gonorrhea, and CRO, and the current last-line treatment for gonorrhea is ineffective against chlamydia (48–52). This situation highlights the urgent need for an oral antibiotic that can target both pathogens simultaneously.

Epetraborole (EBO) is a boron-containing compound that inhibits protein synthesis in bacteria by inhibiting the leucyl-tRNA synthetase (LeuRS) (53). The drug passed phase 1 in clinical trials with a high safety profile in humans and was evaluated in both Phase II and Phase III clinical trials for the treatment of refractory *Mycobacterium avium* complex (MAC) lung disease. However, new patient enrollment in the Phase III trial was paused following a preliminary review of Phase II data, which raised concerns regarding the drug’s efficacy against MAC (54–58). The aim of this study is to assess the effectiveness of EBO against the most prevalent bacterial STIs, *C. trachomatis* and *N. gonorrhoeae*. We evaluated the anti-gonococcal and anti-chlamydial activity of EBO against pathogenic strains. Moreover, we assessed its killing activity against *N. gonorrhoeae* through a time-kill assay and its ability to inhibit *C. trachomatis* inclusion maturation. Additionally, the safety profile was assessed via cytotoxicity on endocervical cells. The ability of EBO to clear both intracellular *N. gonorrhoeae* and *C. trachomatis* inclusions was evaluated by *in vitro* co-infection model. Finally, the *in vivo* efficacy of EBO was evaluated in a mouse model of *N. gonorrhoeae* genital tract infection.

## MATERIALS AND METHODS

### Bacterial strains, media, and reagents

*N. gonorrhoeae* strains were obtained from the CDC, the WHO, and the American Type Culture Collection (ATCC) (Table S1). The cell lines, including the HEC-1B (HTB-113), the ME-180 (HTB-33), and the McCoy (CRL-1696), were purchased from ATCC. Antibiotics were obtained commercially: gentamicin (Chem-Impex International, Wood Dale, IL, USA), gepotidacin (GEP) (Ambeed, Arlington Heights, IL, USA), and streptomycin (STP), as well as CRO and AZM (TCI America, Portland, OR, USA). Media and reagents were obtained from commercial vendors: McCoy’s 5A medium, hematin, and Eagle’s Minimum Essential Medium (MEM) (Sigma Aldrich, St. Louis, MO, USA), Difco GC base medium, de Man, Rogosa and Sharpe (MRS) broth, chocolate II agar plates, brucella broth, bovine hemoglobin and IsoVitaleX (Becton Dickinson & Company, Cockeysville, MD, USA), triton X-100 (Acros Organics, Fair Lawn, NJ, USA), nicotinamide adenine dinucleotide (NAD) (Chem-Impex International, Wood Dale, IL, USA), the *C. trachomatis* major outer membrane protein (MOMP) primary and secondary antibody (Bio-Rad, Hercules, CA, USA), MTS (BOC Sciences, Shirley, NY, USA), Hoechst 33342 (MedChemExpress, Monmouth Junction, NJ, USA), and the phalloidin conjugates California red (AAT-Bioquest, Pleasanton, CA, USA).

### Antibacterial activity of EBO against *N. gonorrhoeae*

The inhibitory activity of EBO and control antibiotics (CRO and AZM) was determined against 16 multidrug-resistant *N. gonorrhoeae* strains using the agar dilution method following the guidelines of the Clinical and Laboratory Standards Institute (CLSI) (59). Briefly, GC agar with hemoglobin was prepared and supplemented with 1% IsoVitalex, and different concentrations of EBO, AZM, and CRO were utilized. To assess the susceptibility, *N. gonorrhoeae* cells were suspended in a phosphate buffer solution (PBS) and diluted to ~10^5^ CFU/mL and spotted on the GC agar plates supplemented with test agents. Plates were incubated at 37°C with 5% CO_2_ for 24 h, and the minimum concentration of the drug that completely inhibited bacterial growth on the surface was considered as the minimum inhibitory concentration (MIC).

### Evaluation of the killing kinetics of EBO against *N. gonorrhoeae*

A time-kill assay experiment was conducted for EBO against *N. gonorrhoeae* FA1090, and *N. gonorrhoeae* CDC-181 (AZM-resistant) to evaluate the killing kinetics of EBO, as previously described (60, 61). Briefly, *N. gonorrhoeae* strains in logarithmic phase were suspended in brucella broth supplemented with 1% IsoVitaleX to a final titer of ~1 × 10^6^ CFU/mL and exposed to either EBO (at 4 × MIC for FA1090 and CDC-181) or positive controls (CRO) (at 4 × MIC for both strains) and AZM (at 4 × MIC for FA1090 and 32 µg/mL for CDC-181). DMSO (the solvent of drugs) was used in this experiment as a negative control. Bacteria were incubated and aliquots were withdrawn at the indicated time points to count the viable bacterial cells on chocolate II agar plates.

### Post-antibiotic effect of EBO against *N. gonorrhoeae*

The post-antibiotic effect of EBO and AZM was evaluated against three *N. gonorrhoeae* strains (FA1090, WHO-Y, and WHO-X) by following the procedure described elsewhere (62, 63). Briefly, brucella supplemented broth was used to grow *N. gonorrhoeae* strains to a log-phase before being diluted in fresh media to ~5 × 10^6^ CFU/mL. Thereafter, the cells were exposed to EBO and AZM at (10 × MIC) for 1 h at 37°C with 5% CO_2_. Cells incubated with DMSO served as the growth control. Next, the drugs were removed by diluting the suspended bacteria 1:500 using fresh media. Afterward, the diluted bacteria were incubated at 37°C with 5% CO_2_. Every 2 h, aliquots were withdrawn and serially diluted for plating onto chocolate II agar plates. Here, we calculated the post-antibiotic effect according to the following equation: post-antibiotic effect = *T*_Sample_ − *T*_Control_, where *T*_Sample_ is the time required for the bacteria to increase 1 log_10_ after removing the antibiotic, and *T*_Control_ is the time needed for a 1 log_10_ increase in the bacterial titer without exposure to the antibiotic.

### Intracellular bacterial clearance assay against *N. gonorrhoeae*

ME-180 cells infected with *N. gonorrhoeae* were used to evaluate the ability of EBO in penetrating and clearing intracellular *N. gonorrhoeae* (64). Briefly, a monolayer of confluent ME-180 cells was infected with *N. gonorrhoeae* FA1090 (MOI of 10) in the same conditions mentioned above. After the incubation time, the wells were washed and then treated with a medium containing 320 µg/mL gentamicin to kill any extracellular bacteria. Then, the PBS was used to remove any remaining extracellular bacteria or gentamicin traces before being treated with 3 ×  MIC of CRO, AZM, EBO, or DMSO (negative control) by washing the cells three times. Thereafter, 0.5% saponin was added to each well for 1 min, with pipetting, to lyse the ME-180 cells and release the intracellular bacteria. The cell lysates were diluted and spotted on chocolate II agar plates to quantify the recoverable intracellular bacteria.

### *In vitro* cytotoxicity assessment

The high safety profile of EBO was assessed using HEC-1B and ME-180 cell lines, as described previously (65). Briefly, HEC-1B and ME-180 cell lines were seeded in 96-well plates using MEM media with 10% FBS and McCoy’s 5A media with the same concentration of FBS for 24 h at 37°C and 5% CO_2_. Subsequently, the cells were incubated with fresh media containing high concentrations of EBO (64–256 µg/mL), in triplicate, under the same conditions for an additional 24 h. Finally, cytotoxicity was assessed by measuring the color change of MTS due to the reduction of NADH in viable cells, as determined by an absorbance reading at 490 nm (OD490).

### Media conditions and propagation of *C. trachomatis*

*C. trachomatis* serovar L2 (ATCC VR-902B) was utilized in this study and propagated using the McCoy cell line as reported previously (66). Briefly, the McCoy cell line was cultured in MEM and incubated at 37°C with 5% CO_2_ until reaching confluency. Then, the MEM medium was changed to fresh media containing *C. trachomatis* (at a multiplicity of infection [MOI] of 1) supplemented with 1 mg/mL of cycloheximide (to reduce protein synthesis for the McCoy cells during the infection without affecting *Chlamydia*) (67). After 2 h of infection, fresh medium without antibiotics was added and incubated at 37°C with 5% CO_2_ for 48–60 h to form inclusions inside the McCoy cells. After the incubation time, the host cells were lysed, and the *Chlamydia* cells were harvested for storage at −80°C. During this study, 3 cell lines were used to confirm the activity of EBO against *C. trachomatis*, including the CDC-recommended cell line, McCoy (68), and the previously reported cell lines, human endocervical epithelial cells (ME-180) and the endometrial epithelial cell line (HEC-1B) (69–73).

### Anti-chlamydial activity investigation

The immunofluorescent assays were conducted to assess the activity of EBO against the *C. trachomatis*-infected McCoy cell line, as reported previously (66, 74). Briefly, McCoy cells were infected with *C. trachomatis* L2 as described above for 2 h before being treated with EBO (4 µg/mL) for 24 h. AZM (4 µg/mL) and CRO (8 µg/mL) were included as controls. After the treatment period, the medium was removed, and the cells were fixed for 15 min, permeabilized for 15 min, and blocked for 60 min using 4% (wt/vol) formaldehyde, 0.5% (vol/vol) Triton X-100, and 1% bovine serum albumin, respectively. The *C. trachomatis* MOMP antibody was incubated with the cells overnight before using the Hoechst 33342, donkey anti-goat IgG conjugated to Alexa 488, and the phalloidin conjugates California red for an hour. The cells were washed between each step to reduce background noise. Finally, the images were visualized using Nikon Eclipse Ti2 inverted microscope (Nikon, Melville, NY, USA).

To assess the exact impact of EBO on *C. trachomatis*, HEC-1B and ME-180 cells were seeded in 96-well plates and then infected with *C. trachomatis* L2 at an MOI of 1 for 2 h before adding different concentrations of EBO (0.25–16 µg/mL) for 48 h at 37°C with 5% CO_2_. AZM (4 µg/mL), CRO, and gepotidacin (GEP) (8 µg/mL) were used as controls. The infected untreated cells were used as a negative control. After the treatment period, the host cells were disrupted to harvest the *C. trachomatis* infectious bodies. The cell lysates were used to reinfect a fresh seeded monolayer of the cell line in a 10-fold dilution series. After another 24 h, the infected cells were fixed and stained as described above to calculate the recoverable infectious progeny. The same experiment was repeated to fix and stain the infected HEC-1B and ME-180 with and without treatment for immunofluorescent imaging.

### *C. trachomatis* reactivation assay

Two sets of each cell line (HEC-1B and ME-180) were infected with *C. trachomatis* and treated with EBO and control antibiotics as described above in the titration and immunofluorescent assays. HEC-1B cell lines were cultured in MEM medium, and McCoy’s 5A medium was utilized for the ME-180 cell line. Briefly, after treating the cells for 24 h, drug-free medium was added to the cells for additional 24 h. Then, the cells were fixed and stained for imaging and subsequently lysed for counting.

### *In vitro N. gonorrhoeae/C. trachomatis* co-infection model

The co-infection model was performed using *N. gonorrhoeae* CDC-181 and *C. trachomatis* following the procedure described previously (44) with modifications. Briefly, the ME-180 cell line was seeded in a 96-well plate for 24 h at 37°C with 5% CO_2_. Thereafter, the medium was changed to a medium containing *C. trachomatis* L2 (MOI = 1) for 2 h after which the medium was replaced with *N. gonorrhoeae* CDC-181 (MOI = 10)-containing medium. The cells were then incubated at 37°C with 5% CO_2_ for 4 h. Thereafter, the cells were washed and incubated with 320 µg/mL gentamicin to kill extracellular bacteria. Then, the wells were washed with PBS before adding media that contained CRO (8 µg/mL), AZM (8 µg/mL), or EBO (8, 4, or 2 µg/mL) for 42 h at 37°C with 5% CO_2_. Finally, the cells were disrupted after washing with PBS to enumerate the *C. trachomatis* and *N. gonorrhoeae* using the spotting and titration assays described above.

### Tracking *C. trachomatis* inclusion development

The HEC-1B cells were cultured and infected with *C. trachomatis* for 16 h as described above. Subsequently, the cells were treated with media containing EBO at a fixed concentration of 16 µg/mL for 8 h. The cells incubated with DMSO served as a negative control for comparison. During treatment, fixed fields revealed non-infectious replicating reticulate bodies within inclusions in both control and treated cells, which were selected and visualized at various time points: 0, 4, and 8 h. The size of inclusions was measured using imageJ 1.53 m.

### The activity of EBO against vaginal microflora

The commensal vaginal bacterial strains, including *Lactobacillus gasseri* and *L. crispatus,* were utilized to assess the effect of EBO on vaginal microflora using the broth microdilution assay following the CLSI guidelines (59, 75). Briefly, lactobacilli were cultured on MRS agar plates and incubated before being suspended in PBS to achieve a 0.5 McFarland standard. The cells were then diluted in MRS broth to obtain ~5 × 10^5^ CFU/mL. Serial dilutions of EBO, CRO, and AZM were incubated with bacteria for 48 h at 37°C with 5% CO_2_.

### *In vivo* efficacy of EBO in a mouse model of *N. gonorrhoeae* vaginal infection

The *in vivo* model was conducted in accordance with procedures approved by the Institutional Animal Care and Use Committee of Virginia Polytechnic Institute and State University. Before starting the model, ovariectomized female BALB/c mice were distributed randomly in cages (5 mice/cage). The efficacy of EBO was evaluated *in vivo* using a mouse model as described elsewhere (65, 76–78). Two days prior to the infection, 8-week-old mice were implanted subcutaneously with a 5 mg sustained-release estradiol pellet for 21 days. A combination of antibiotics was used to disrupt the normal vaginal microbiota to enhance gonococcal colonization, consisting of vancomycin (0.6 mg) and STP (1.2 mg), which was injected intraperitoneally from the day of pellet insertion to 1 day after the infection in addition to supplementing the drinking water with trimethoprim (0.4 g/L). Thereafter, STP (5 g/L) was added to the trimethoprim in the drinking water and maintained throughout the experiment. Two days after pellet implementation, mice were infected with CRO-resistant *N. gonorrhoeae* WHO-X (77) (3 × 10^6^ CFU/mouse) intravaginally and left for 2 days to allow the bacterial colonization. Thereafter, mice received treatments as follows: one group received the vehicle as a negative control, one group was treated with EBO (25 mg/kg orally twice daily for two consecutive days), and the third group was treated with CRO (15 mg/kg/once intraperitoneally). The samples were collected daily from the vagina using sterile cotton swabs. Bacteria were enumerated after spotting the 10-fold dilution series on GC agar supplemented with nystatin, vancomycin, colistin, and trimethoprim.

### Statistical analyses

Statistical analyses were conducted using GraphPad Prism 9.0 (GraphPad Software, La Jolla, CA, USA). Several statistical methods, including one-way and two-way ANOVA with a post hoc Dunnett’s test, were utilized to evaluate the significance and measure the *P*-values. The *in vitro* assays were performed in triplicate (three biological replicates for each). The data are presented as means ± standard deviation. The standard deviation was calculated from these biological replicates means. For all data, ns represents the non-significance, * refers to a significance with *P*-value < 0.05, ** stands for a significant result with *P*-value < 0.01, and **** is a significant result with *P*-value < 0.0001.

## RESULTS

### The anti-gonococcal activity of EBO

**Fig 1:**
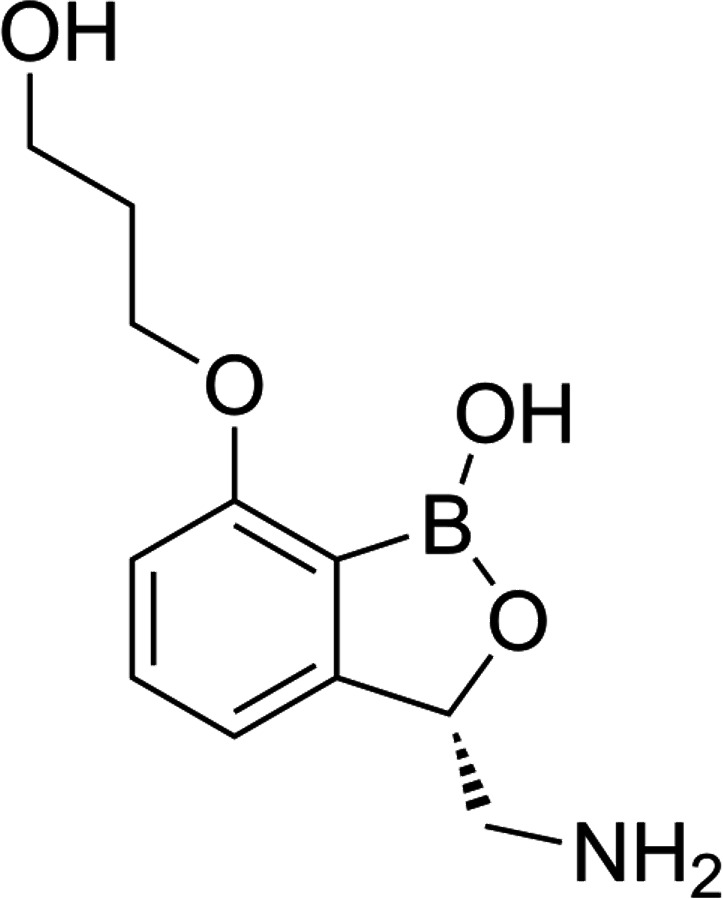
The chemical structure of EBO.

The susceptibility analysis of EBO (Fig. 1) was rigorously evaluated against 16 multidrug-resistant *N. gonorrhoeae* isolates, which have a wide range of resistance profiles, using the agar dilution method. This included WHO reference strains that have well-established phenotypic and genetic markers (79, 80). EBO exhibited potent inhibitory activity against the 16 multidrug-resistant *N. gonorrhoeae* strains with MICs ranging from 0.125 to 0.25 µg/mL, inhibiting 50% of the strains (MIC_50_) at 0.125 µg/mL and 90% of the strains tested (MIC_90_) at 0.25 µg/mL (Table 1). Interestingly, EBO maintained the same activity against strains exhibiting high resistance levels to the drug of choice for treating gonorrheal infections, CRO, as seen in strains WHO-Z, WHO-Y, and WHO-X in addition to strains with high resistance levels to the recommended drug for treating chlamydial infection, AZM, such as WHO-V and CDC-181 (Table 1; Table S1).

**TABLE 1:** The MICs (μg/mL) of EBO and control antibiotics against 16 multidrug-resistant *N. gonorrhoeae* isolates^*a*^

*Neisseria gonorrhoeae* strains	EBO	AZM	CRO
FA 1090	0.125	0.125	0.004
CDC-166	0.125	0.5	0.032
CDC-167	0.25	16	0.016
CDC-171	0.125	0.5	0.032
CDC-172	0.125	1	0.032
CDC-173	0.125	1	0.064
CDC-174	0.125	2	0.064
CDC-175	0.25	8	0.004
CDC-179	0.25	8	0.016
CDC-181	0.25	>128	0.032
CDC-182	0.125	1	0.032
CDC-202	0.25	16	0.004
WHO-V	0.25	>128	0.064
WHO-X	0.125	0.5	2
WHO-Y	0.25	0.5	1
WHO-Z	0.25	0.5	0.5
MIC_50_	0.125	1	0.032
MIC_90_	0.25	>128	1

^*a*^ EPO, epetraborole; AZM, azithromycin; CRO, ceftriaxone.

### Time-kill kinetics of EBO against multidrug-resistant *N. gonorrhoeae*

**Fig 2:**
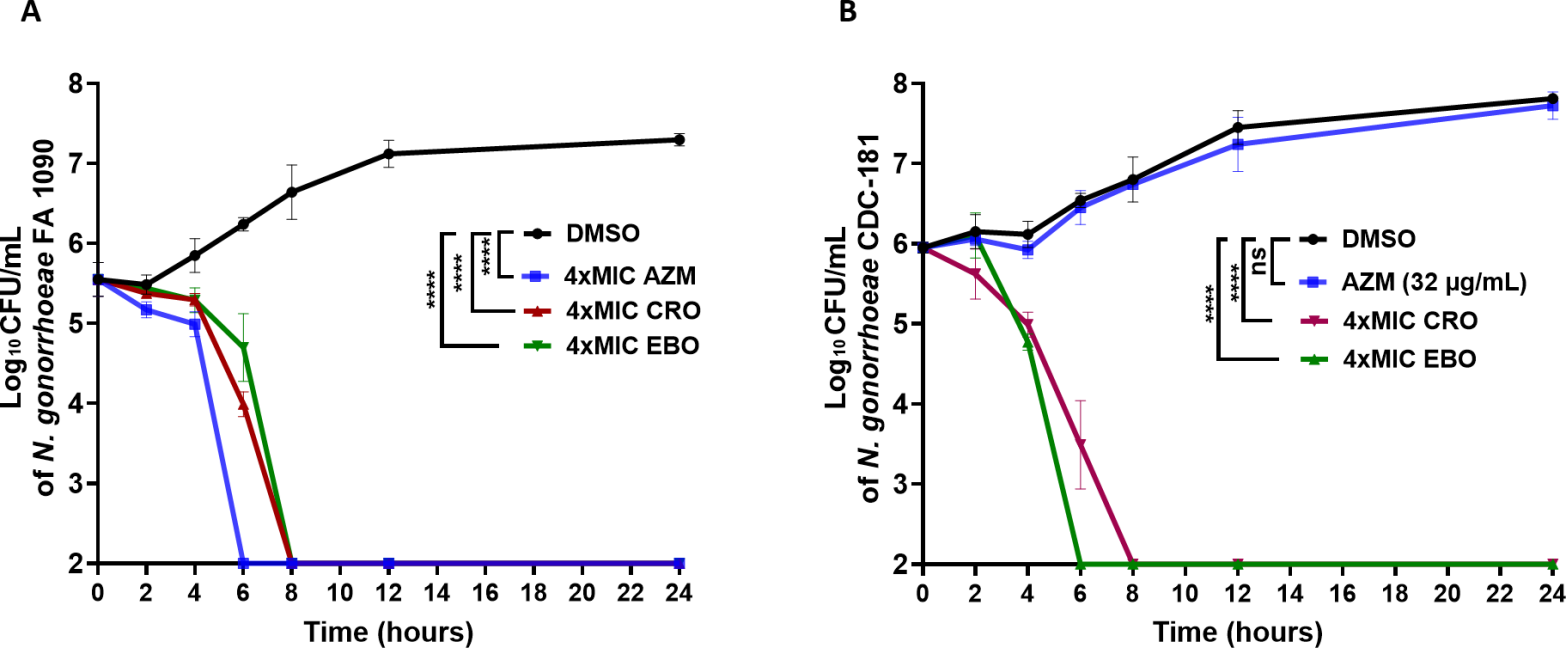
Time-kill kinetics of EBO against *N. gonorrhoeae. N. gonorrhoeae* FA1090 (**A**) or CDC-181 (**B**) were incubated with EBO, CRO, AZM (at the indicated concentrations), or DMSO (as a negative control), and samples were enumerated at time points of 0, 2, 4, 6, 8, 12, and 24 h. Each point is presented as the average of log_10_ CFU/mL, and error bars represent the with standard deviation for the untreated group (DMSO), epetraborole (EBO), ceftriaxone (CRO), and azithromycin (AZM). The significant differences between the groups were analyzed via two-way ANOVA. Asterisks (****) reveal significant differences (*P* < 0.0001, and ns stands for non-significance). The experiment was performed in triplicate (three biological replicates for each). The standard deviation (SD) was calculated from these biological replicates means.

The killing kinetics of EBO were assessed against AZM-resistant *N. gonorrhoeae* CDC-181 and AZM-susceptible *N. gonorrhoeae* FA1090 using a standard time-kill assay. As depicted in Fig. 2A and B, EBO (at 4 × MIC) displayed a bactericidal activity against *N. gonorrhoeae* FA1090 and CDC-181, completely eliminating the high inoculum of *N. gonorrhoeae* within 6–8 h. While AZM exerted a bactericidal activity against *N. gonorrhoeae* FA1090, clearing the bacterial load after 6 h (Fig. 2A), it has no activity (at 32 µg/mL) against *N. gonorrhoeae* CDC-181 (Fig. 2B). CRO (4 × MIC) required 8 h to completely clear *N. gonorrhoeae* FA1090 and CDC-181 counts. These results highlight the potential of the EBO to demonstrate rapid bactericidal activity, representing a notable advantage in combating gonorrheal infection.

### Post-antibiotic effect of EBO against multidrug-resistant *N. gonorrhoeae*

After confirming the bactericidal activity of EBO against *N. gonorrhoeae*, we assessed the post-antibiotic effect of EBO after a brief exposure to bacterial cells. We found that EBO, similar to AZM, exhibited modest post-antibiotic inhibitory activity (4 h) after treating *N. gonorrhoeae* cells for 1 h (Table 2), which was comparable to the effect of AZM (post-antibiotic effect = 6–8 h).

**TABLE 2:** Post-antibiotic effect (in hours) of epetraborole (EBO) and azithromycin (AZM) against *N. gonorrhoeae*

*N. gonorrhoeae* strains	EBO	AZM
FA1090	4	6
WHO-Y	4	6
WHO-X	4	8

### Intracellular clearance activity of EBO against *N. gonorrhoeae*

**Fig 3:**
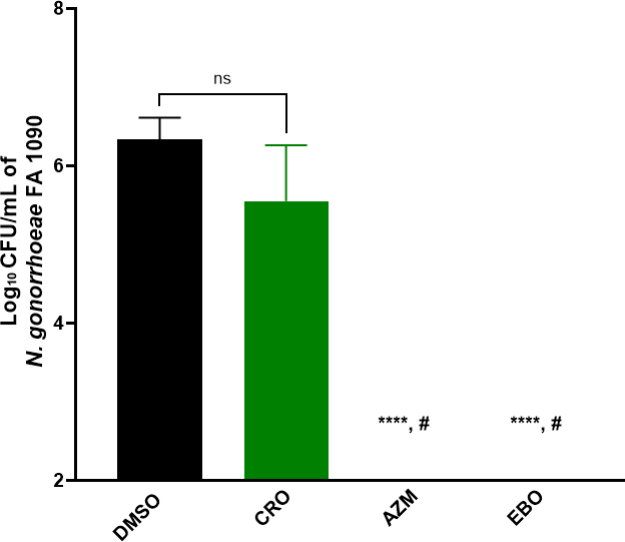
The intracellular clearance activity of epetraborole (EBO) (at 3 × MIC) against ME-180 cells infected with *N. gonorrhoeae* FA1090. DMSO is used as a negative control, and azithromycin (AZM) and ceftriaxone (CRO) were included as controls. Asterisks (****) reveal significant differences (*P* < 0.0001) between the EBO and AZM relative to DMSO (untreated), and a pound sign (#) indicates a significant difference between EBO and AZM in comparison to CRO. The experiment was performed in triplicate (three biological replicates for each). The standard deviation (SD) was calculated from these biological replicates means.

*N. gonorrhoeae* can penetrate and colonize endocervical cells, enabling the bacteria to survive intracellularly and disseminate infection. Therefore, drugs that can clear the bacteria intracellularly are desirable. Thus, the intracellular clearance assay was conducted to evaluate the activity of EBO in eliminating the intracellular *N. gonorrhoeae*. As depicted in Fig. 3, EBO, at 3× MIC, eradicated the burden of intracellular *N. gonorrhoeae* infection inside ME-180 cells after 24 h of incubation, which was similar to the activity of AZM. Interestingly, EBO was superior to CRO, which failed to significantly reduce the bacterial burden. These findings indicate that EBO can eliminate *N. gonorrhoeae* more effectively than the drug of choice for *N. gonorrhoeae* infections, CRO.

### The safety profile of EBO against ME-180 and HEC-1-B cells

Cytotoxicity data were collected for EBO to assess its tolerability to human cells. Against the ME-180 cell line, EBO exhibited no cytotoxicity at the high concentration of 256 µg/mL (Fig. S1A). Likewise, EBO exhibited a high safety profile with no cytotoxicity against Fig. S1B). The high potency of EBO against multidrug-resistant bacteria *N. gonorrhoeae* (MICs = 0.125–0.25 µg/mL) alongside its low cytotoxicity (>256 µg/mL) provides a wide safety window of more than 1,000-fold, subsequently, suggesting a favorable selectivity index for potential human use.

### The anti-chlamydial activity of EBO

Since EBO demonstrated potent activity against multidrug-resistant *N. gonorrhoeae,* including strains resistant to CRO and AZM, we sought to investigate whether it possesses a similar potency against *C. trachomatis*. Immunofluorescent staining was utilized to determine the MIC of EBO against *C. trachomatis* serovar L2. In this study, AZM at a fixed concentration of 4 µg/mL, which is the minimum chlamydicidal concentration (MCC) in human cell lines such as HL and HEP-2, was utilized as a positive control (81). Additionally, infected cells without treatment (DMSO) or treated with CRO at a high concentration of 8 µg/mL were used as negative controls. First, the activity of EBO was evaluated by immunofluorescence assay after treating McCoy cell lines with EBO (4 µg/mL) for 2 h post-infection with *Chlamydia* L2, followed by a 48 h incubation. After analyzing the results, EBO completely inhibited the growth of *C. trachomatis* L2 and was used for further investigations.

**Fig 4:**
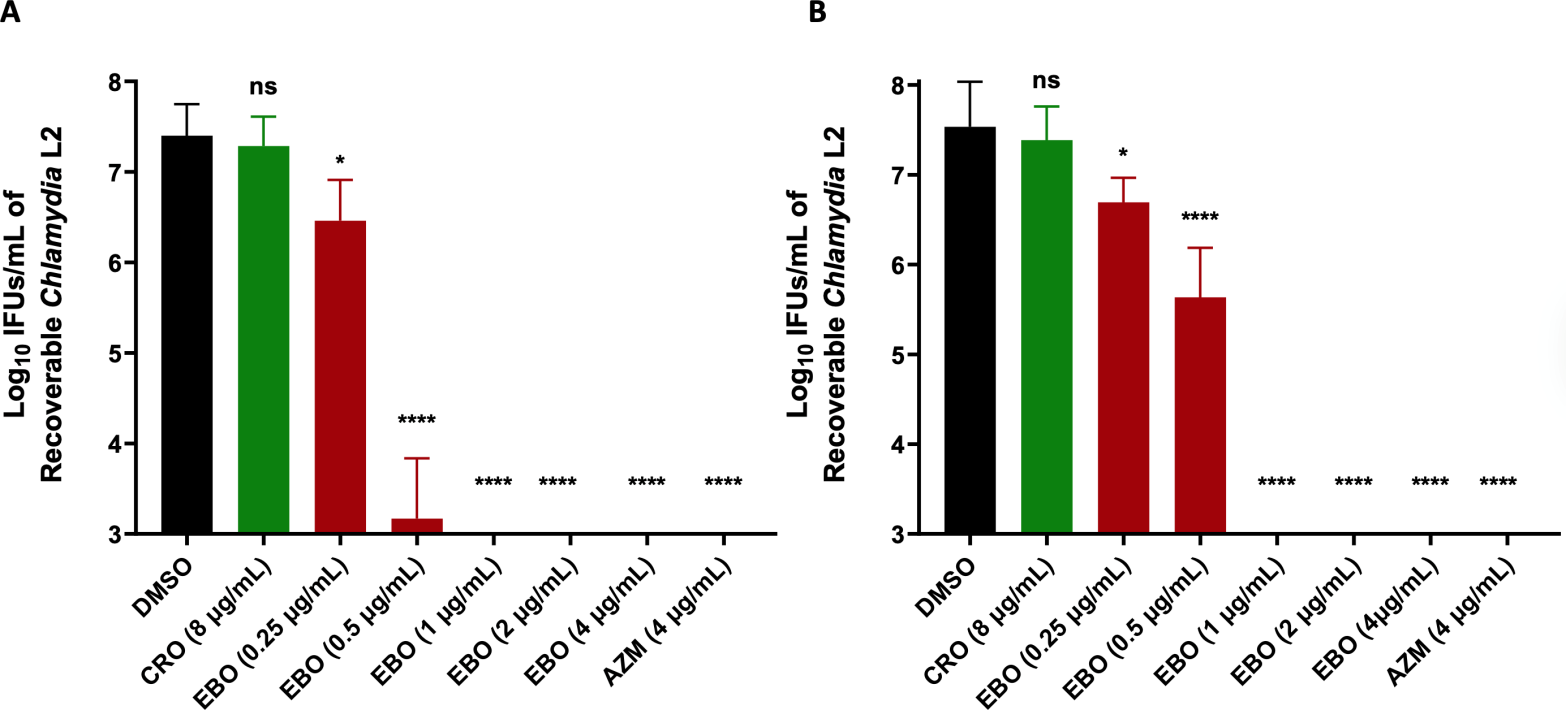
Quantification of *Chlamydia* L2 infectious progeny yield after treating the infected: (**A**) ME-180 cells or (**B**) HEC-1-B cells with EBO and control antibiotics compared to untreated cells. The timeframe of treatment is 48 h post-infection with *Chlamydia* L2 for 2 h. The results are reported on a log_10_ scale of the mean ± standard deviation (shown as an error bar) for the untreated group (DMSO), ceftriaxone (CRO), epetraborole (EBO), and azithromycin (AZM). Asterisks (*) and (****) reveal significant differences (*P* < 0.05 and *P* < 0.0001, respectively), and ns stands for non-significance after using a one-way ANOVA test. The experiment was performed in triplicate (three biological replicates for each). The standard deviation (SD) was calculated from these biological replicates means.

Next, we investigated the drug’s anti-chlamydial activity at different concentrations. As depicted in Fig. 4, *C. trachomatis* serovar L2 was completely attenuated when treated with EBO or AZM at concentrations of 1 and 4 µg/mL, respectively. Moreover, a significant reduction of 4.3 log_10_ and 1.9 log_10_ in the infection yield was observed in comparison with the untreated group after using 0.5 µg/mL of EBO in HEC-1B and ME-180 cells, respectively. Additionally, the lower concentration of EBO (0.25 µg/mL) has a significant effect against *C. trachomatis,* resulting in a reduction of more than 1 log_10_ CFU (>90% reduction) (Fig. 4). However, no significant difference was found between CRO (8 µg/mL) and DMSO.

Microscopic images of ME-180 (Fig. S2) and Hec-1-B (Fig. S3) cells infected with *C. trachomatis* serovar L2 revealed that treatment with EBO and AZM clearly impacted the size and development of the bacterial inclusions. After incubating EBO at 0.5 µg/mL with endocervical cell lines infected with *C. trachomatis* for 48 h, the bacterial inclusions were significantly diminished. Moreover, treatment with EBO or AZM at concentrations of 1 and 4 µg/mL completely blocked the development of *C. trachomatis* inclusion bodies.

**Fig 5:**
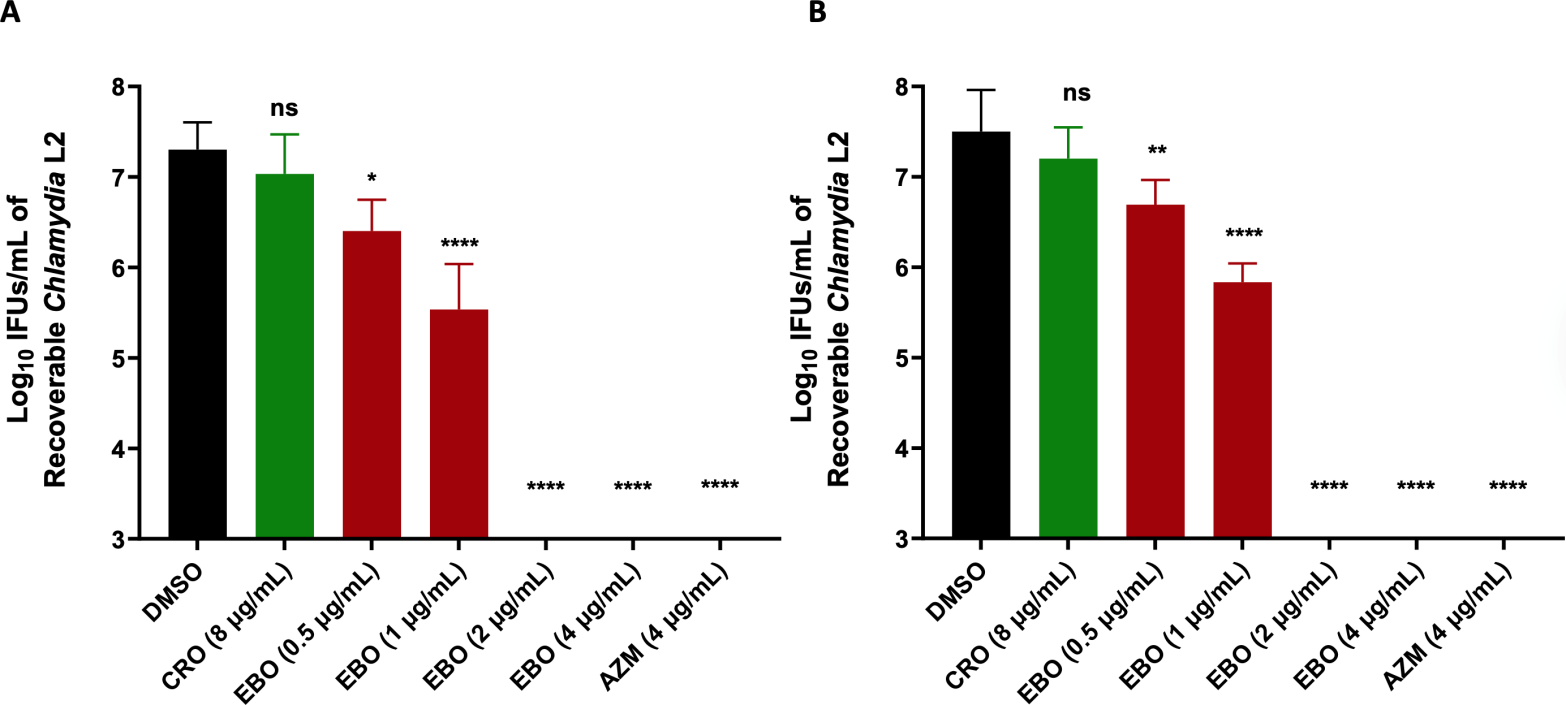
Enumeration of *C. trachomatis* L2 infectious progeny yield after reactivating the infected: (**A**) ME-180 or (**B**) HEC-1-B cells treated with EBO and control antibiotics. The timeframe of treatment is 24 h post-infection with *Chlamydia* L2 for 2 h, followed by another 24 h of recovery. The results are reported on a log_10_ scale, with the mean ± standard deviation for the untreated group (DMSO), ceftriaxone (CRO), epetraborole (EBO), and azithromycin (AZM). Asterisks (*), (**), and (****) reveal significant differences (*P* < 0.05, *P* < 0.01, and *P* < 0.0001, respectively), and ns stands for non-significance after using a one-way ANOVA test. The experiment was performed in triplicate (three biological replicates for each). The standard deviation (SD) was calculated from these biological replicates means.

Next, we investigated the effect of EBO on the reactivation of *C. trachomatis* after infecting cells and incubating them with drug-free media for recovery. Then, the cells were disrupted to determine the bacterial count using the standard IFU protocol. AZM, at a concentration of 4 µg/mL, was used as a positive control. Additionally, the infected cells without treatment or CRO at 8 µg/mL served as negative controls. The inclusion count illustrates that EBO acts as a bactericidal agent against *C. trachomatis* at 2 and 4 µg/mL. Moreover, at a lower concentration of 1 µg/mL, EBO exhibited a bactericidal effect by blocking chlamydial development to a 1.8 log_10_ and 1.7 log_10_ lower level than the untreated cells in ME-180 and HEC-1B, respectively (Fig. 5A and B). Additionally, severe attenuation of recoverable infectious progeny was observed with AZM (4 µg/mL), and no activity was detected for CRO against *C. trachomatis* L2. Additionally, immunofluorescence images of inclusions after treating cells with EBO show alterations in size and morphology compared to those of untreated cells (Fig. S4 and S5).

### Activity of EBO in an *in vitro N. gonorrhoeae*-*C. trachomatis* co-infection model

**Fig 6:**
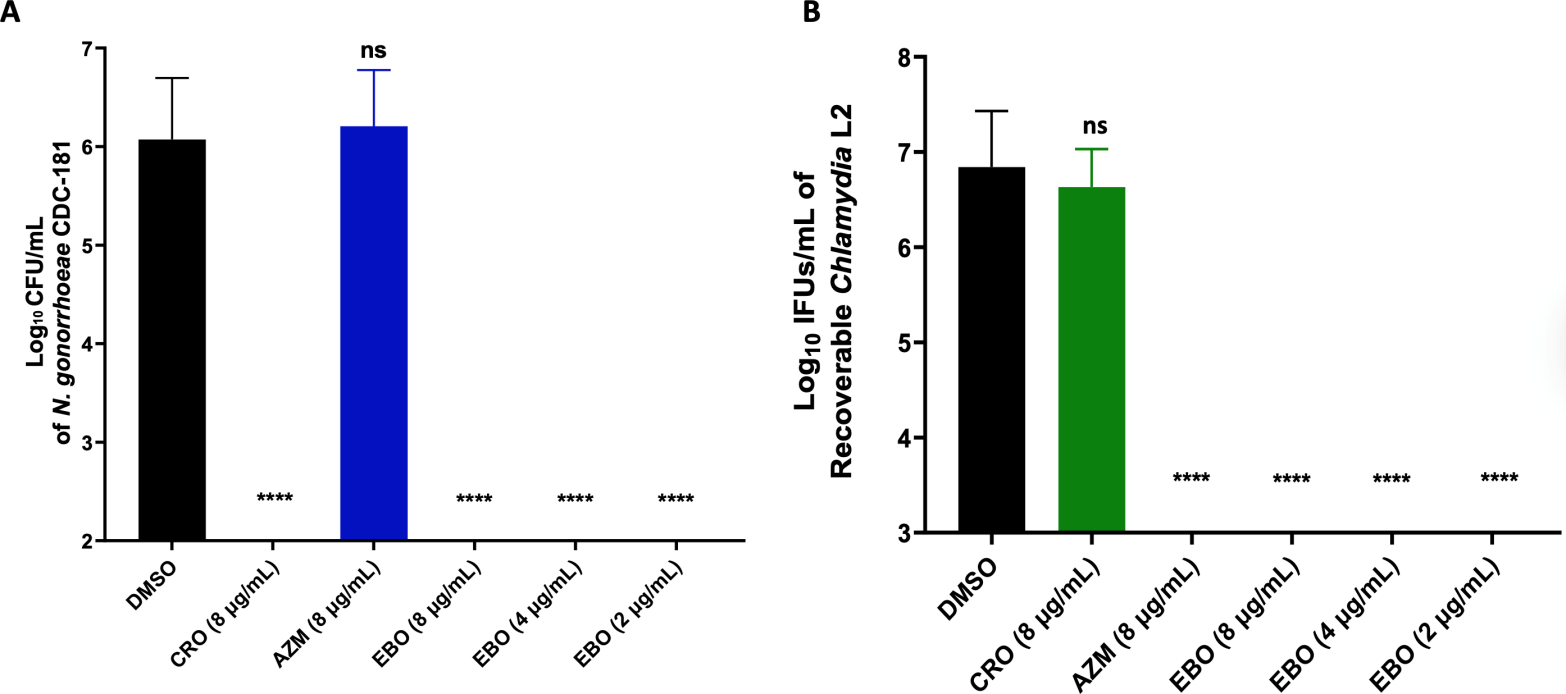
The intracellular clearance activity of epetraborole (EBO) in an *in vitro N. gonorrhoeae-C. trachomatis* co-infection model. ME-180 cells were infected with both *N. gonorrhoeae* CDC-181 and *C. trachomatis* L2 for 6 h and treated with epetraborole (EBO) (at 2, 4, and 8 µg/mL), ceftriaxone (CRO) (at 8 µg/mL), or azithromycin (AZM) (at 8 µg/mL). The data are presented as average (**A**) log_10_ CFU/mL and (**B**) log_10_ IFU/mL of *N. gonorrhoeae* CDC-181, and *C. trachomatis* L2, respectively. Error bars represent standard deviation values. Asterisks (****) reveal significant differences (*P* < 0.0001) between test agents relative to DMSO (untreated). The experiment was performed in triplicate (three biological replicates for each). The standard deviation (SD) was calculated from these biological replicates means.

Our previous observations of a significant reduction in *N. gonorrhoeae* and *C. trachomatis* titers inside infected cells after treating them with EBO encouraged us to evaluate the potential antibacterial activity of EBO using an *in vitro* co-infection model. During this analysis, the experimental conditions were applied to support the growth of both *N. gonorrhoeae* and *C. trachomatis*, and cells were treated with EBO (2, 4, and 8 µg/mL), as well as AZM and CRO (8 µg/mL). As shown in Fig. 6, EBO, at all tested concentrations, was capable of clearing *N. gonorrhoeae* intracellularly and inhibiting the formation of inclusions by *C. trachomatis*. Remarkably, EBO was superior to CRO, which acted effectively only against *N. gonorrhoeae* at a high concentration of 8 µg/mL (1,000-fold higher than its MIC), without any activity against *C. trachomatis* at this concentration. Likewise, EBO was superior to AZM, which inhibited the inclusion formation of *C. trachomatis* but had no activity against *N. gonorrhoeae* CDC-181 at a high concentration of 8 µg/mL.

### EBO inhibited *C. trachomatis* inclusion maturation

**Fig 7:**
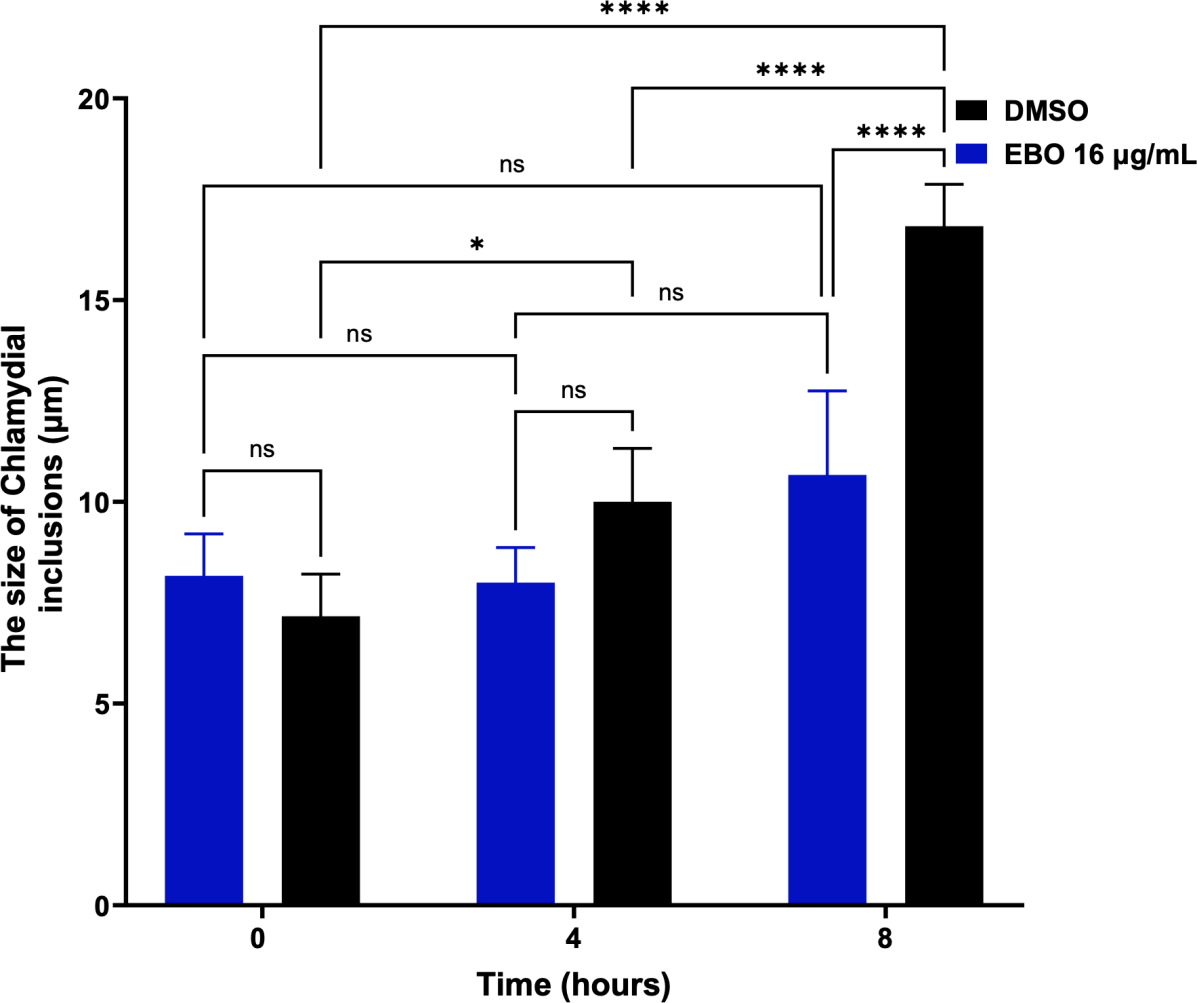
Evaluation of the effect of epetraborole (EBO) on the development of chlamydial inclusion after infecting Hec-1B cell line with *C. trachomatis* L2 for 16 h. Each point represents the average size of inclusions in μm measured using imageJ, for the untreated group (DMSO) and epetraborole (EBO). Error bars represent the standard deviation values. The measurements were taken at time points of 0, 4, and 8 h after treatment. Asterisks (*) and (****) reveal significant differences (*P* < 0.05 and *P* < 0.0001, respectively), and ns stands for non-significance. The experiment was performed in triplicate (three biological replicates for each). The standard deviation (SD) was calculated from these biological replicates means.

To monitor the activity of EBO against well-developed replicating reticulate bodies after they form inclusions, we developed a novel live single-cell analysis technique. HEC-1-B cells were infected with *C. trachomatis* L2 at an MOI of 1 for 16 h and then treated with EBO at a fixed concentration of 16 µg/mL, and cells with drug-free medium were used as a negative control. The coordinates of randomly infected cells were selected and used to image the infected cells and evaluate the activity of EBO. As presented in Fig. S6, utilizing EBO significantly inhibited the replication and enlargement of the inclusion size compared to the negative control. Moreover, the development of inclusions was measured using imageJ. As presented in Fig. 6, the changes in the inclusion sizes after using EBO (at a concentration of 16 µg/mL) were not significant from 0 to 4 h, 0 to 8 h, and 4 to 8 h. Conversely, the inclusion size of the negative control (DMSO) changed significantly from 7.13 to 10 µm after 4 h. Additionally, a significant increase in size occurred after 8 h, reaching 16.8 µm, which is more than double the original size (Fig. 7).

### Susceptibility analysis of EBO against vaginal microbiome

The healthy vaginal microbiota protects the body from colonization by *N. gonorrhoeae* and *C. trachomatis*. In the genitourinary tract, different species of lactobacilli have a significant role in preventing colonization of pathogenic *N. gonorrhoeae* and *C. trachomatis*. Thus, we assessed the effectiveness of EBO against four vaginal lactobacilli isolates. EBO demonstrated lower activity compared to control drugs (MIC ≥ 16 µg /mL) (Table 3). Conversely, CRO, the drug of choice for *N. gonorrhoeae,* and AZM, the recommended treatment for *C. trachomatis,* exhibited potent activity in inhibiting the growth of representative microbiota isolates (MICs ≤ 0.5 µg/mL).

**TABLE 3:** MICs (µg/mL) of epetraborole (EBO) against vaginal normal microflora strains^*a*^

Strains	EBO	AZM	CRO
*Lactobacillus gasseri* HM 404	32	≤0.5	≤0.5
*Lactobacillus gasseri* HM 407	32	≤0.5	≤0.5
*Lactobacillus gasseri* HM 641	16	≤0.5	≤0.5
*Lactobacillus crispatus* HM 422	16	≤0.5	≤0.5

^*a*^ EBO, epetraborole; AZM, azithromycin; CRO, ceftriaxone.

### *In vivo* efficacy of EBO in a mouse model

**Fig 8:**
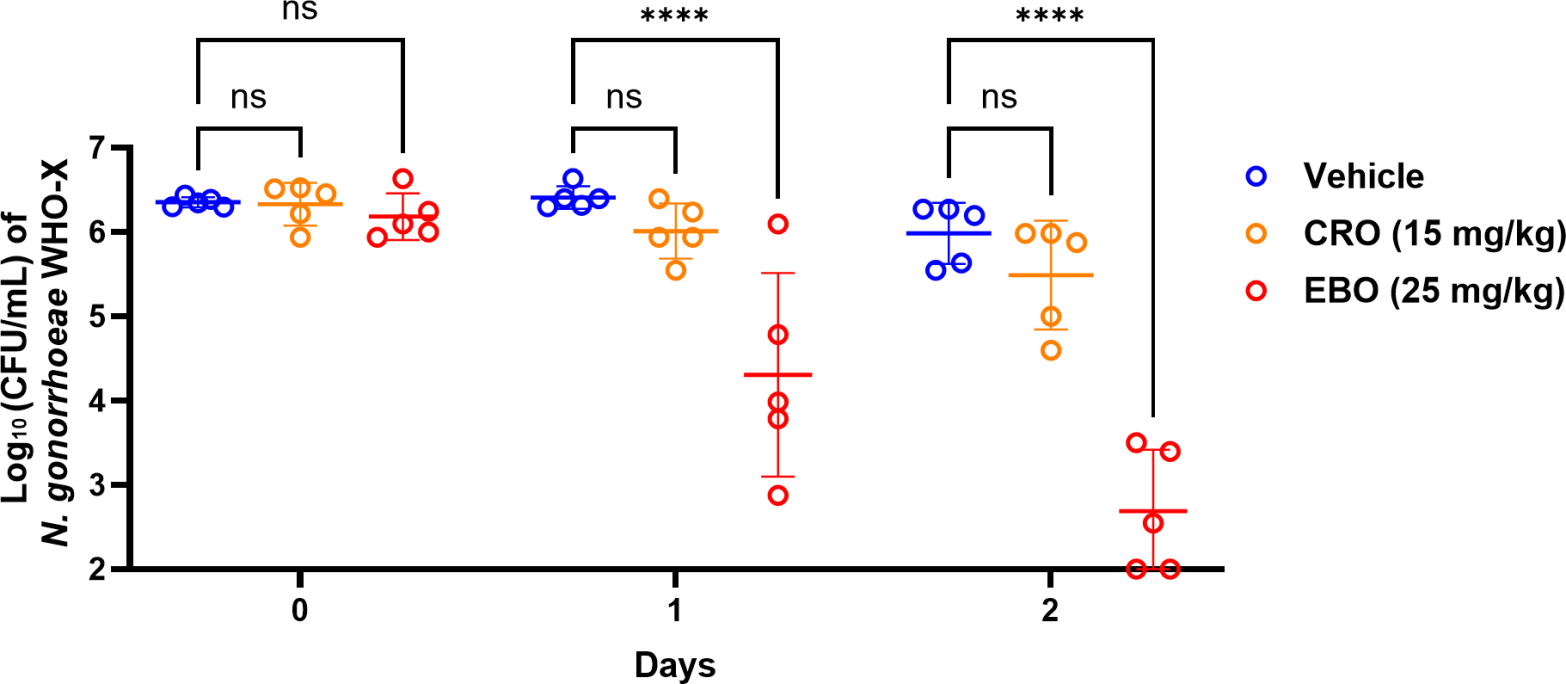
The *in vivo* efficacy of epetraborole (EBO) against *N. gonorrhoeae* WHO-X (ceftriaxone-resistant) after treatment for 2 days. Estradiol-implanted mice were infected with the CRO-resistant *N. gonorrhoeae* WHO-X. Mice were administered EBO (25 mg/kg/day; *n* = 5) orally for two consecutive days. As a control, a single dose of CRO (15 mg/kg; *n* = 5) was administered intraperitoneally to treat a group of mice. Each point represents the average log_10_ CFU/mL, with standard deviation, for the untreated group (vehicle; *n* = 5), ceftriaxone (CRO), and epetraborole (EBO). A two-way ANOVA test was used to analyze the results and determine their significance. Asterisks (****) denote a significant difference (*P* < 0.0001) between EBO relative to the vehicle (untreated) and ns stands for non-significance.

Finally, the efficacy of EBO (at 25 mg/kg/day orally for two consecutive days) was evaluated in female ovariectomized BALB/c mice infected intravaginally with CRO-resistant *N. gonorrhoeae* WHO-X. After 2 days of treatment, EBO resulted in a significant reduction in the bacterial load, generating ~3.3 log_10_ CFU reduction, which corresponds to a reduction of 99.95% in the bacterial count compared to the vehicle (Fig. 8). On the other hand, CRO (15 mg/kg/once intraperitoneally) showed a slight reduction in the burden of *N. gonorrhoeae* in mice infected with the CRO-resistant strain WHO-X. The bacterial counts in the mice administered the vehicle remained unchanged during the study, indicating that the significant reduction in *N. gonorrhoeae* observed in mice treated with EBO is attributed to the drug treatment.

## DISCUSSION

Sexually transmitted infections (STIs) pose a major global health challenge, exemplified by the more than 1 million curable cases acquired worldwide each day (6). This burden is further compounded by the fact that *N. gonorrhoeae* and *C. trachomatis* infections are frequently asymptomatic, resulting in underdiagnosis and detection of only a fraction of their true prevalence (82–84). Additionally, there is no single treatment that effectively addresses co-infections with *C. trachomatis* (85). Given that no effective vaccines are currently available for use in clinical practice to prevent *C. trachomatis* or *N. gonorrhoeae*, the development of new effective drugs is essential and a priority (86, 87). Through the repurposing of drugs and compounds in clinical trials, we identified EBO as a promising new candidate for treating STIs. EBO is a benzoxaborole derivative that showed potent activity against clinical *Mycobacterium abscessus* isolates (88). In 2022, EBO completed a phase 1b clinical trial study (NCT04892641), which evaluated 28-day oral dosing regimens in healthy volunteers (55). Thus, the data regarding its safety and pharmacokinetics are known in humans.

Herein, the antibacterial activity of EBO was evaluated against a panel of 16 clinical isolates of *N. gonorrhoeae* using the agar dilution method. EBO successfully inhibited the bacterial growth of *N. gonorrhoeae* strains tested at low concentrations, with MIC_50_ and MIC_90_ values of 0.125 and 0.25 µg/mL, respectively. However, AZM and CRO showed lower effectiveness against multidrug-resistant *N. gonorrhoeae* with MIC_90_ values of >128 and 1 µg/mL, respectively. The MIC results of the control antibiotics were in agreement with previous reports (89). Additionally, EBO maintained the same activity against strains that are resistant to several antibiotics, including tetracycline, STP, penicillin, ciprofloxacin, AZM, CRO, and cefixime. Additionally, EBO was superior to GEP in *in vitro* anti-gonococcal activity against the panel of 6 WHO reference strains of *N. gonorrhoeae* with different resistance profiles. EBO inhibited the tested strains at concentrations ranging from 0.125 to 0.25 µg/mL, while GEP MICs ranged from 0.5 to 8 µg/mL (Table S2). Interestingly, EBO maintained its potent activity against GEP-sensitive strains (WHO-X, WHO-Y, and WHO-Z) (MIC values = 0.125–0.25 µg/mL) and against the strains with reduced susceptibility to GEP (WHO-G, WHO-L, and WHO-M) (MIC values = 0.25 µg/mL) (Table S2).

Interestingly, after analyzing the anti-chlamydial activity of EBO and the control antibiotics, such as CRO and GEP, we found that 8 µg/mL of the control antibiotics is insufficient to inhibit the chlamydial inclusions in ME-180 cells. We confirmed the anti-chlamydial activity of EBO using two different cell lines, HEC-1B and ME-180, where it exhibited a significant effect on the size and number of the inclusions after using low concentrations.

One of the alternative life cycles in *Chlamydia* is persistence, which allows the organism to escape the host immune response (90). To evaluate the ability of EBO to eliminate elementary bodies and prevent persistence, a reactivation assay was utilized against *C. trachomatis* (74). Our findings align with a previous report showing that the activity of EBO at 1 µg/mL blocks chlamydial growth after 48 h of treatment (91). However, a reactivation assay indicated that using the same concentration of EBO at 1 µg/mL did not prevent chlamydial growth, which is consistent with previous research (91). Therefore, complete attenuation of recoverable infectious progeny was observed at higher concentrations of EBO (2 µg/mL). Additionally, immunofluorescence images of EBO at lower concentrations showed a dramatic change in inclusion size and morphology compared to those of the untreated ones. Our results on the antibacterial activity of EBO against *N. gonorrhoeae* and *C. trachomatis* are in agreement with previous reports that have shown the antibacterial activity of EBO against Gram-negative bacteria, with an MIC range of 0.25–4 µg/mL (53, 92). Additionally, the ineffectiveness of CRO and GEP, used as controls in this study, against *C. trachomatis* is coincident with previous reports (52, 93). Moreover, AZM was utilized as a positive control in all of the experiments due to its known activity against *C. trachomatis* (74).

The time-kill kinetics assay demonstrated the bactericidal activity of EBO against *N. gonorrhoeae* FA1090 after exposure to 4 × MIC, which eliminated the bacterial cells within 8 h. The killing time for EBO is the same as that of CRO, the current drug of choice for gonorrhea. AZM exhibited a rapid bactericidal activity against FA1090, which is consistent with a previous report (77). A bactericidal antibiotic is preferred over a bacteriostatic one for quelling infections and preventing transmission, which are desirable characteristics for new anti-*N*. *gonorrhoeae* therapeutics (94).

The post-antibiotic effect has a significant role in determining the dosing frequency for new drugs (95). *N. gonorrhoeae* exhibited a 4 h delay in growth compared to the control after exposure to EBO for an hour, which was comparable to that of AZM. AZM exhibited a post-antibiotic effect of 6–8 h, which is in agreement with previous reports (62, 96, 97).

Developing an *in vitro* STI co-infection model provides a relevant experimental model for cases infected with both *N. gonorrhoeae* and *Chlamydia,* allowing for the evaluation of the antibacterial activity of potential drugs. *N. gonorrhoeae* and *C. trachomatis* invade mammalian cells in the female genital tract, reside inside them, replicate, and transfer from cell to cell (9, 98). New therapeutics capable of eliminating the burden of intracellular *N. gonorrhoeae* and *C. trachomatis* are needed. CRO, the drug of choice for *N. gonorrhoeae* infections, was unable to eradicate chlamydia and eliminated only *N. gonorrhoeae* intracellularly at high concentrations. The limited intracellular clearance activity of CRO can be attributed to the complex and bulky structure of CRO and bacterial aggregates (99, 100); it also lacks activity against *Chlamydia* (101). EBO exhibited a significant reduction against the resistant strain of *N. gonorrhoeae* along with *C. trachomatis* after following the *in vitro* coinfection model reported previously (44).

A healthy vaginal microbiome dominated by *Lactobacillus* species is less susceptible to STIs, including *N. gonorrhoeae* and *C. trachomatis* (102–104). A disadvantage of the standard-of-care antibiotics used for treating *N. gonorrhoeae* and *C. trachomatis* infections is that they indiscriminately inhibit the growth of beneficial commensal bacteria (102–104). We studied the activity of EBO against *Lactobacillus* species, which are members of the healthy microbiota in the female urogenital tract. We found that EBO has a limited inhibitory effect on the growth of commensal *Lactobacillus* spp., with a safety window of more than 64-fold compared to its activity against *N. gonorrhoeae* and *C. trachomatis*.

The emergence and rapid spread of CRO-resistant *N. gonorrhoeae* represent a serious global health concern, as CRO remains the last effective option for empiric gonococcal treatment. In recent years, CRO-resistant clones have been increasingly reported, particularly in Asia, where resistance is associated mainly with the penA-60.001 allele. Resistance is defined by isolates exhibiting a MIC greater than 0.125 mg/L and alarming escalation of such resistant strains between 2017 and 2022 (105). Even more concerning, strains exhibiting both ceftriaxone resistance and high-level azithromycin resistance have been reported worldwide (31). These trends underscore the urgent need for novel therapeutic agents that are effective against resistant strains. In this context, we evaluated the efficacy of EBO against the CRO-resistant *N. gonorrhoeae* strain WHO-X using an *in vivo* mouse model for gonococcal infection (77). While, CRO showed no activity against *N. gonorrhoeae* WHO-X *in vivo*, showing no significant difference from the untreated group, EBO demonstrated potent activity achieving a 99.95% reduction in bacterial burden after just 2 days of treatment. Taken together, these findings highlight the potential of EBO as a first-in-class oral therapeutic for the treatment of drug-resistant gonorrhea and chlamydial infections. Further clinical evaluation will be essential to advance EBO from a promising candidate to an effective, patient-ready therapy.

### Conclusion

To conclude, we report that EBO was effective against multidrug-resistant *N. gonorrhoeae* and *C. trachomatis in vitro*. EBO exhibited a bactericidal activity and a prolonged post-antibiotic effect against *N. gonorrhoeae*. Moreover, EBO showed a high safety profile against mammalian cell lines (ME-180 and HEC-1B), and it exhibited limited activity against the beneficial vaginal lactobacilli. Additionally, EBO was superior to CRO and AZM in a co-infection model. Moreover, EBO demonstrated significant *in vivo* efficacy, achieving a 99.95% reduction in bacterial burden against the CRO-resistant *N. gonorrhoeae* WHO-X. Collectively, our study suggests that EBO represents a promising therapeutic agent against pathogens causing STIs, which merits further investigation.

## SUPPLEMENTAL MATERIAL

Supplemental material aac.01445-25-s0001.docx Tables S1 and S2; Fig. S1 to S8.

ASM does not own the copyrights to Supplemental Material that may be linked to, or accessed through, an article. The authors have granted ASM a non-exclusive, world-wide license to publish the Supplemental Material files. Please contact the corresponding author directly for reuse.
