# Supplementary material for: Repurposing epetraborole to combat Neisseria gonorrhoeae and Chlamydia trachomatis infections
Source: Antimicrob Agents Chemother. 2025 Dec 5;70(1):e01445-25. doi: 10.1128/aac.01445-25 (PMC12777565; doi:10.1128/aac.01445-25)
Supplement: Supplemental material — Tables S1 and S2; Fig. S1 to S8. [file aac.01445-25-s0001.docx]

**Table S1. Bacterial strains used in this study and their characterization.**

| ***Neisseria gonorrhoeae* strains** | **Resistance profile** |
| --- | --- |
| FA 1090 | Streptomycin |
| CDC-166 | Tetracycline, penicillin, and ciprofloxacin |
| CDC-167 | Azithromycin |
| CDC-171 | Tetracycline, penicillin, and ciprofloxacin |
| CDC-172 | Tetracycline, penicillin, and ciprofloxacin |
| CDC-173 | Tetracycline, penicillin, and ciprofloxacin |
| CDC-174 | Tetracycline, penicillin, and ciprofloxacin |
| CDC-175 | Azithromycin |
| CDC-179 | Azithromycin |
| CDC-181 | Tetracycline and azithromycin |
| CDC-182 | Tetracycline, penicillin, and ciprofloxacin |
| CDC-202 | Azithromycin |
| WHO-G | Tetracycline and ciprofloxacin. |
| WHO-L | Tetracycline and ciprofloxacin, ceftriaxone and penicillin |
| WHO-M | Tetracycline, ciprofloxacin, and penicillin. |
| WHO-V | Tetracycline, ciprofloxacin, penicillin, and azithromycin |
| WHO-X | Tetracycline, ciprofloxacin, penicillin ceftriaxone and cefixime |
| WHO-Y | Tetracycline, ciprofloxacin, ceftriaxone and cefixime |
| WHO-Z | Tetracycline, ciprofloxacin, penicillin, ceftriaxone and cefixime |

**Table S2:** **The MICs (μg/mL) of EBO, AZM, CRO, and GEP against 6 reference strains.**

| ***Neisseria gonorrhoeae* strains** | **EBO** | **AZM** | **CRO** | **GEP** |
| --- | --- | --- | --- | --- |
| WHO-G | 0.25 | 0.25 | 0.008 | 2 |
| WHO-L | 0.25 | 1 | 0.25 | 8 |
| WHO-M | 0.25 | 0.5 | 0.016 | 2 |
| WHO-X | 0.125 | 0.5 | 2 | 0.5 |
| WHO-Y | 0.25 | 0.5 | 1 | 0.5 |
| WHO-Z | 0.25 | 0.5 | 0.5 | 0.5 |


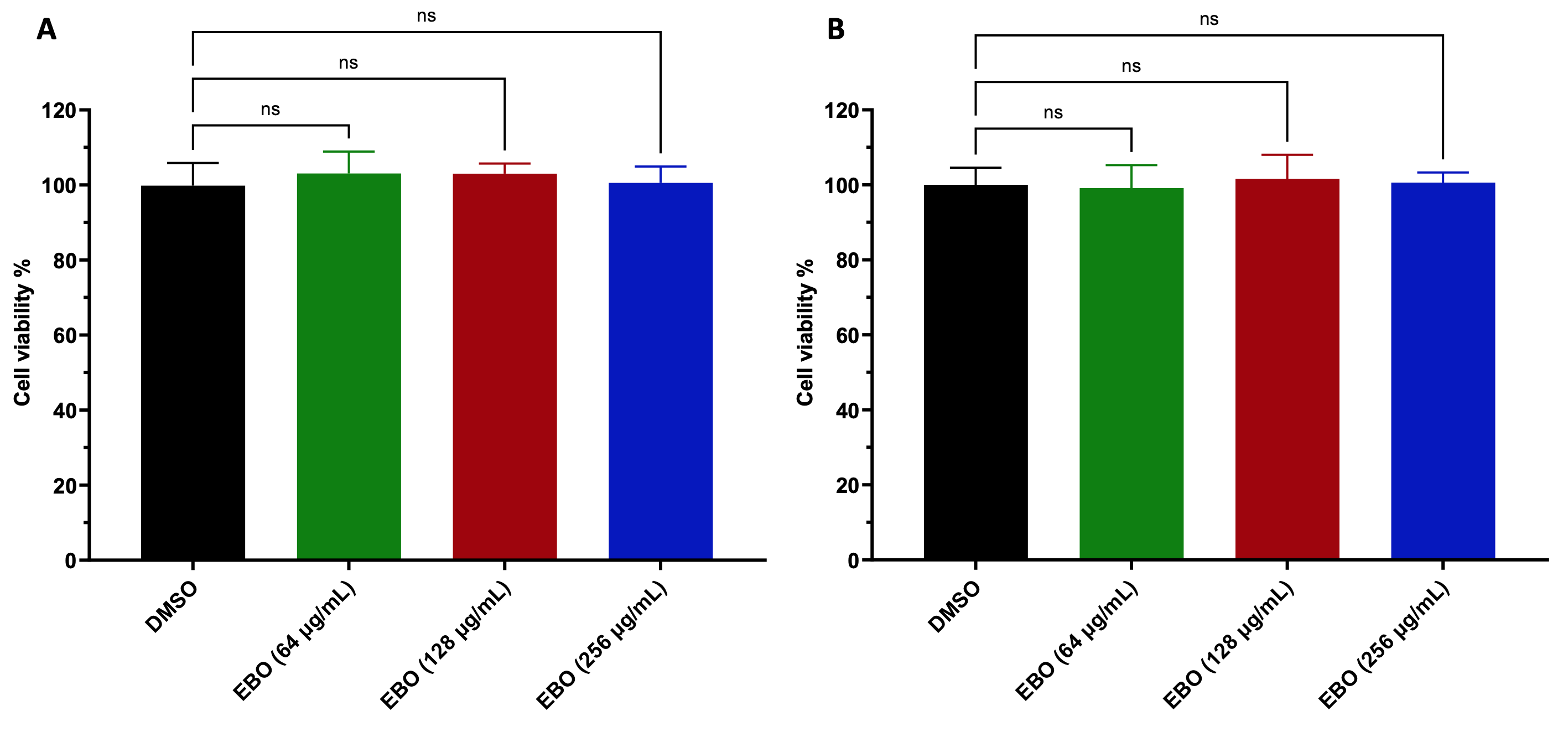
**Figure S1. Cell viability of: A) HEC-1-B, and B) ME-180 cells, after incubation with high concentrations (64, 128, and 256 μg/mL) of epetraborole (EBO) for 24 hours.** Results of cell viability were presented as a percentage viability relative to the untreated cells (DMSO). The one-way ANOVA with post-hoc Dunnett’s test illustrates no significant difference in cell viability between EBO and DMSO.


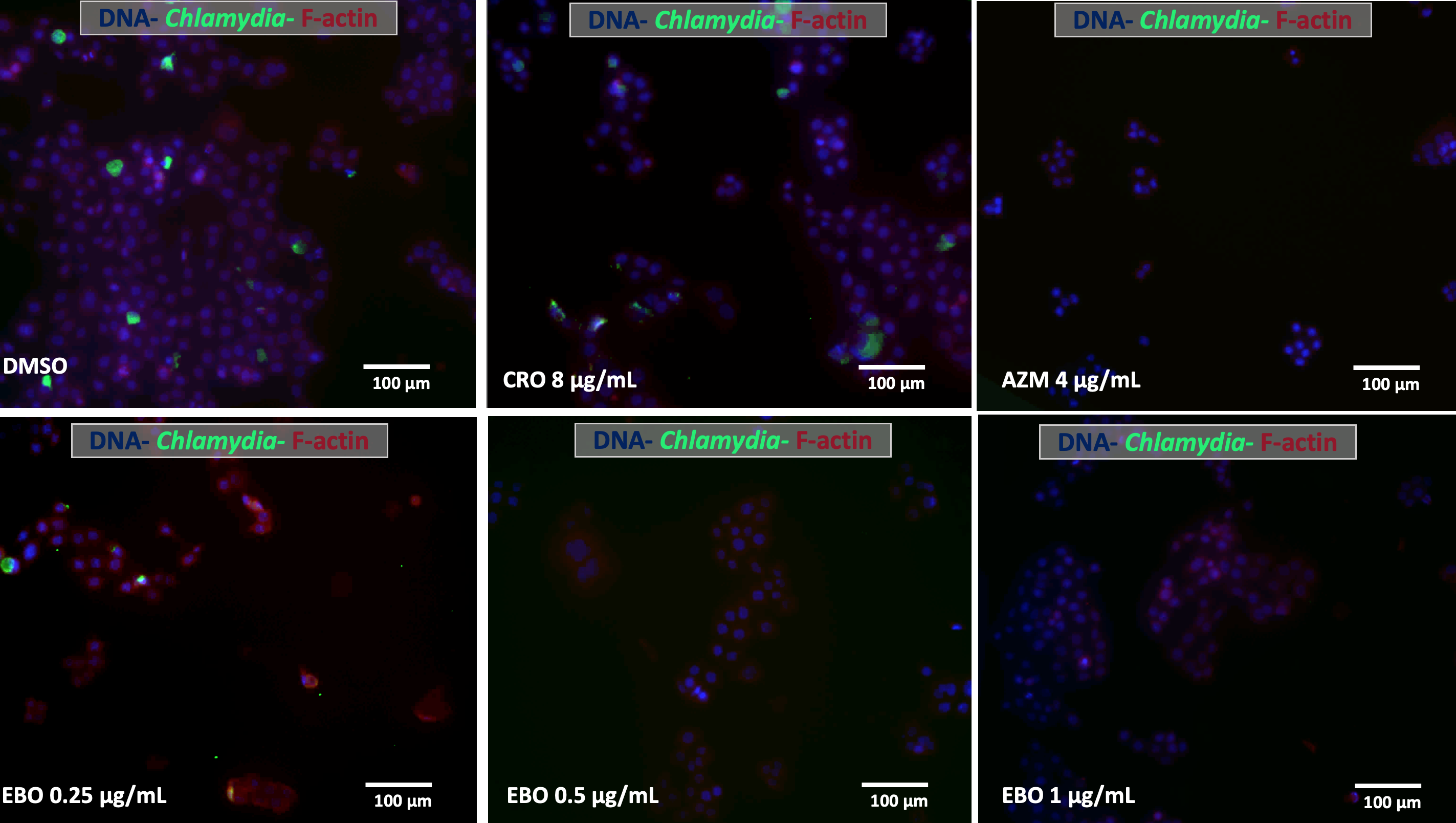


**Figure S2. Immunofluorescence images represent the potential effect of epetraborole (EBO) at different concentrations (0.25, 0.5, and 1 μg/mL) on ME-180 cells infected with *Chlamydia***. The ME-180 cells were infected for 2 hours before being treated with EBO for 48 hours. Azithromycin (AZM) at 4 μg/mL was used as a positive control, and ceftriaxone (CRO) at 8 μg/mL was used as a negative control. The untreated cell (DMSO) was used for comparison of the reduction in morphology and size of the inclusions. The scale bar is 100 μm. Nuclear DNA was stained with Hoechst (blue), the primary antibodies bound to the MOMPs of the chlamydia were stained with donkey anti-goat IgG conjugated to Alexa 488 (green), and the Phalloidin Conjugates California Red was used to stain the F-actin (red).


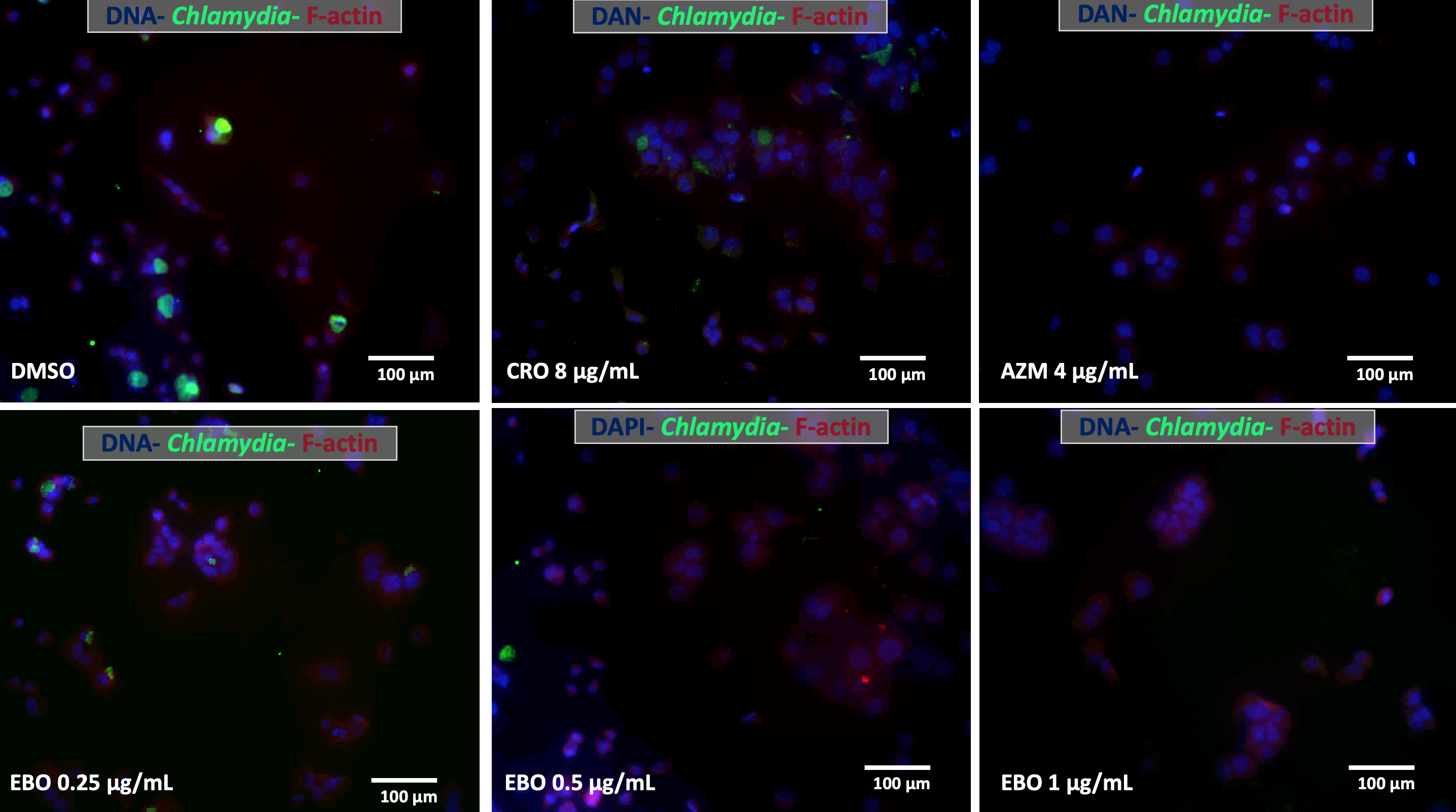


**Figure S3. Immunofluorescence images represent the potential effect of epetraborole (EBO) at different concentrations (0.25, 0.5, and 1 μg/mL) on HEC-1B infected with *Chlamydia*.** The HEC-1B cells were infected for 2 hours and then treated with EBO for an additional 48 hours. Azithromycin (AZM) at 4 μg/mL was used as a positive control, and ceftriaxone (CRO) at 8 μg/mL was used as a negative control. The untreated cell (DMSO) was used for comparison of the reduction in morphology and size of the inclusions. The scale bar is 100 μm. Nuclear DNA was stained with Hoechst (blue), the primary antibodies bound to the MOMPs of the chlamydia were stained with donkey anti-goat IgG conjugated to Alexa 488 (green), and the Phalloidin Conjugates California Red was used to stain the F-actin (red).


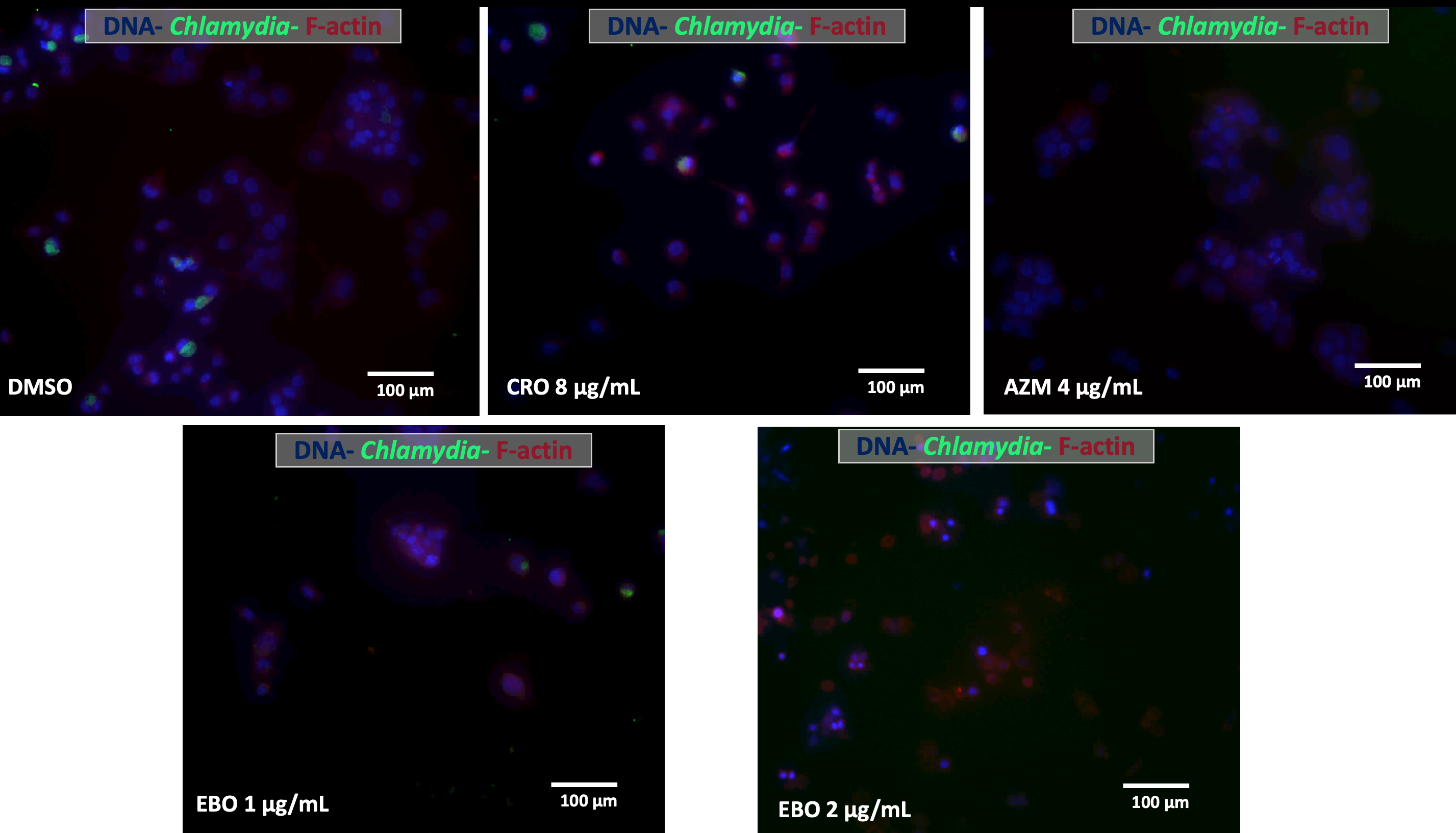


**Figure S4. Immunofluorescence images represent the potential effect of epetraborole (EBO) at different concentrations (1 and 2 μg/mL) on ME-180 infected with *Chlamydia*.** The ME-180 cells were infected for 2 hours before being treated with EBO for 22 hours. The infected cells were then incubated with drug-free media for recovery and assessment for another 24 hours. Azithromycin (AZM) at 4 μg/mL was used as a positive control, and ceftriaxone (CRO) at 8 μg/mL was used as a negative control. The untreated cell (DMSO) was used for comparison of the reduction in morphology and size of the inclusions. The scale bar is 100 μm. Nuclear DNA was stained with Hoechst (blue), the primary antibodies bound to the MOMPs of the chlamydia were stained with donkey anti-goat IgG conjugated to Alexa 488 (green), and the Phalloidin Conjugates California Red was used to stain the F-actin (red).


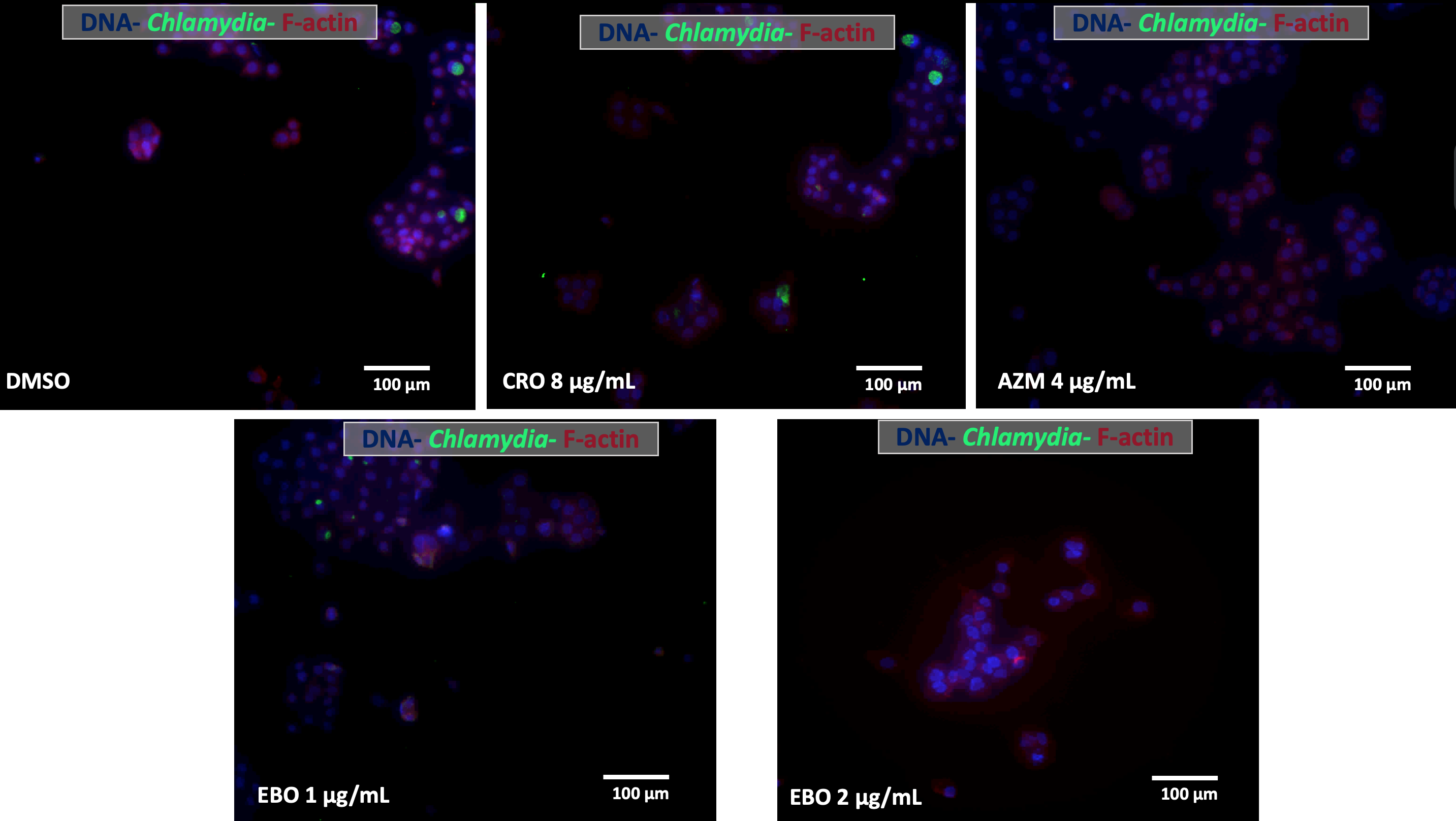


**Figure S5. Immunofluorescence images represent the potential effect of epetraborole (EBO) at different concentrations (1 and 2 μg/mL) on HEC-1B infected with *Chlamydia*.** The HEC-1B cells were infected for 2 hours and then treated with EBO for an additional 22 hours. The infected cells were then incubated with drug-free media for recovery and assessment for another 24 hours. Azithromycin (AZM) at 4 μg/mL was used as a positive control, and ceftriaxone (CRO) at 8 μg/mL was used as a negative control. The untreated cell (DMSO) was used for comparison of the reduction in morphology and size of the inclusions. The scale bar is 100 μm. Nuclear DNA was stained with Hoechst (blue), the primary antibodies bound to the MOMPs of the chlamydia were stained with donkey anti-goat IgG conjugated to Alexa 488 (green), and the Phalloidin Conjugates California Red was used to stain the F-actin (red).


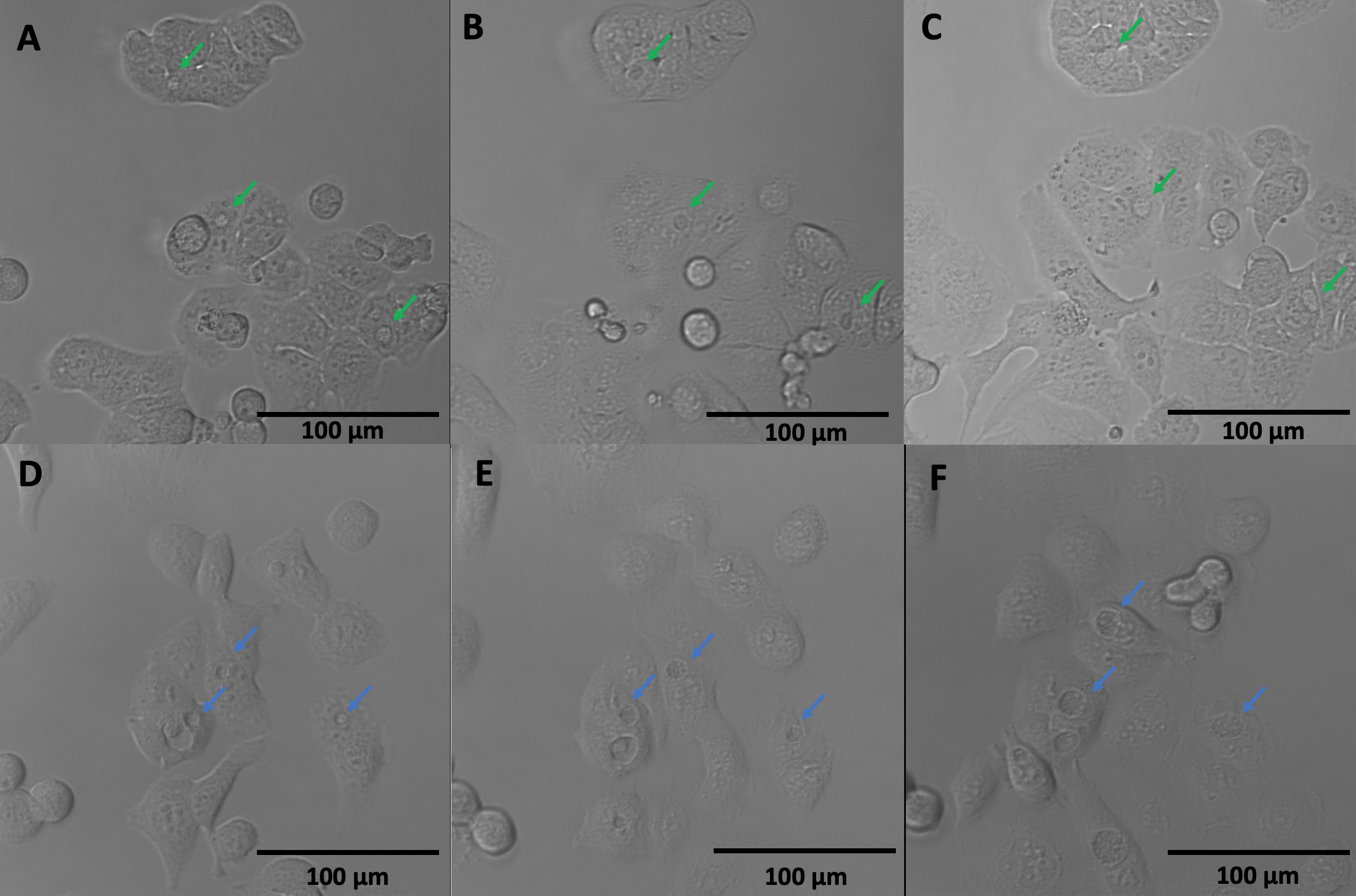


**Figure S6. Evaluation of epetraborole (EBO) effect on the development of chlamydial inclusion after infecting HEC-1B cell line with *C. trachomatis* L2 for 16 hours.** (A-C) represent cells after being treated with EBO at a concentration of 16 μg/mL. (D-F) The untreated group was used for comparison of the development of inclusion over time. (A and D) represent the cells at time point zero, (B and E) after 4 hours from the treatment, or incubated without treatment, (D and F) after 8 hours from the treatment or incubated without treatment. The blue arrows show the development of chlamydial inclusions without treatment. The green arrows depict the static effect of EBO against reticulate bodies.


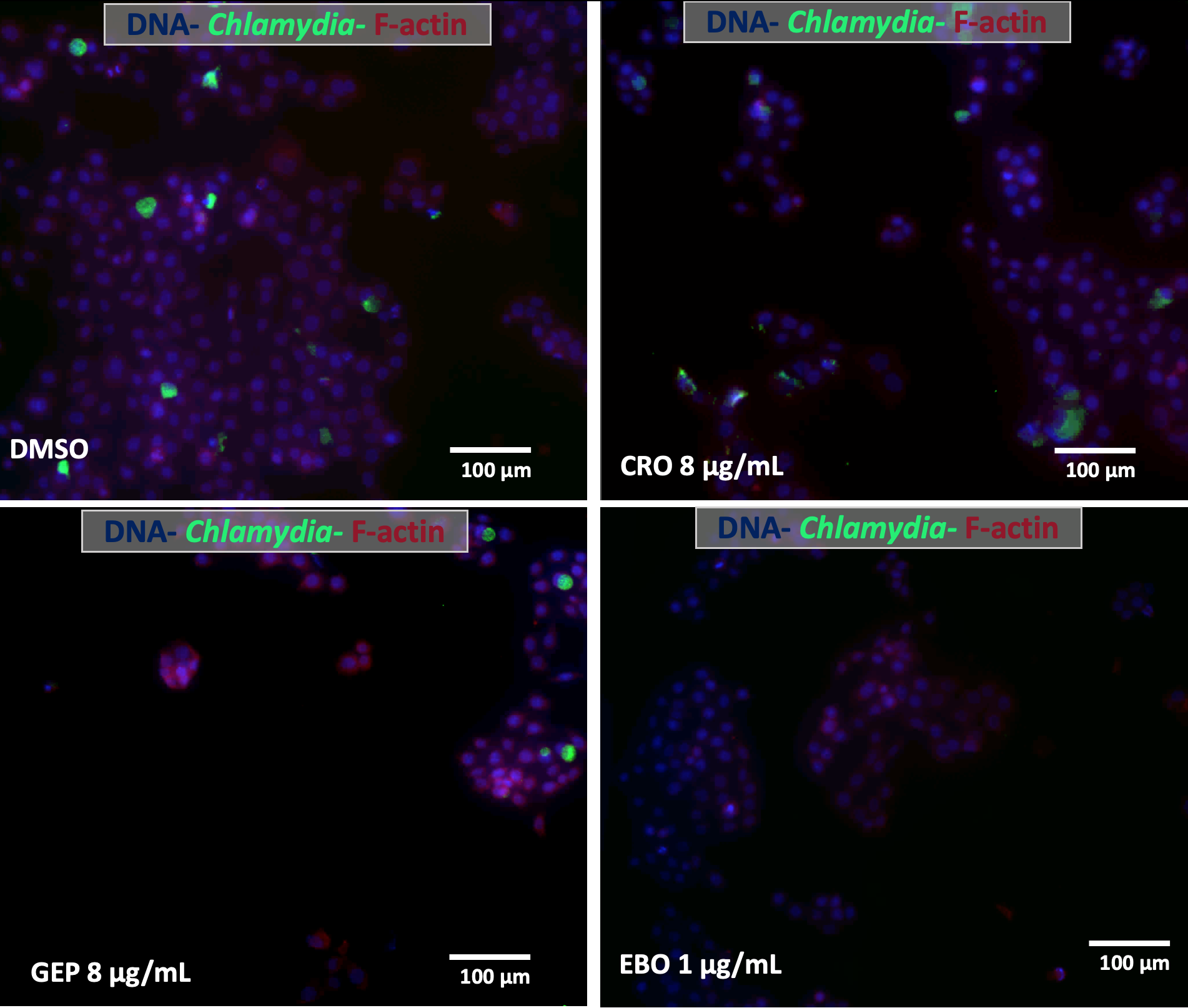


**Figure S7. Immunofluorescence images represent the potential effect of epetraborole (EBO) at 1 μg/mL on ME-180 infected with *Chlamydia*.** The ME-180 cells were infected 2 hours before being treated with EBO for 48 hours. Potential drugs against *N. gonorrhoeae* currently on the market or in clinical trials, such as ceftriaxone (CRO) at 8 μg/mL and gepotidacin (GEP), were evaluated at the same concentration. The untreated cell (DMSO) was used for comparison of the reduction in morphology and size of the inclusions. The scale bar is 100 μm. Nuclear DNA was stained with Hoechst (blue), the primary antibodies bound to the MOMPs of the chlamydia were stained with donkey anti-goat IgG conjugated to Alexa 488 (green), and the Phalloidin Conjugates California Red was used to stain the F-actin (red).

**Figure S8: Enumeration of *Chlamydia* L2 infectious progeny yield in the infected ME-180 cells treated with epetraborole and control antibiotics.** The timeframe of treatment is 48 hours post-infection with *Chlamydia* L2 for 2 hours. The results are reported on a log_10_ scale, with the mean ± standard deviation and error bars for the untreated group (DMSO), ceftriaxone (CRO), epetraborole (EBO), and gepotidacin (GEP). For each graph, transformed values were analyzed by one-way ANOVA with a post hoc Dunnett’s test. Significance values for each sample compared to the untreated control are shown on the graph. ns = not significant and **** = p < 0.0001.
